# The Pharmacogenomics of Opioid Response in Cancer Pain: From Receptor Polymorphisms to Tumour-Mediated Interference—A Narrative-Critical Review

**DOI:** 10.3390/ijms27157011

**Published:** 2026-08-04

**Authors:** Sebastiano Mercadante

**Affiliations:** Main Regional of Supportive/Palliative Care, La Maddalena Cancer Center, Via San Lorenzo 312, 90146 Palermo, Italy; terapiadeldolore@lamaddalenanet.it or 03sebelle@gmail.com

**Keywords:** pharmacogenomics, opioids, cancer pain, OPRM1, CYP2D6, COMT, ABCB1, SLC22A1, membrane transporters, tumour microenvironment, SNP, interindividual variability, precision medicine

## Abstract

Cancer pain affects more than half of all oncology patients and represents one of the most challenging therapeutic problems in modern medicine. Despite the central role of opioids in cancer pain management—codified by the WHO analgesic ladder—the clinical response to these agents is highly variable between individuals. Pharmacogenomics promises to explain this heterogeneity by identifying functionally relevant genetic polymorphisms in genes encoding opioid receptors, metabolizing enzymes, and membrane transport proteins. However, after two decades of intensive research, clinical translation of genetic data into actionable prescribing strategies remains elusive. A narrative-critical review of the literature was conducted by searching PubMed, Scopus, and Web of Science databases from inception to March 2026, using the search terms “opioid,” “pharmacogenomics,” “pharmacogenetics,” “cancer pain,” “polymorphism,” and individual gene names (OPRM1, CYP2D6, COMT, ABCB1, SLC22A1, UGT2B7, and others). Reference lists of included articles were hand-searched for additional relevant studies. Inclusion was restricted to English-language articles reporting original data or systematic reviews on genetic polymorphisms and opioid response, with priority given to studies conducted in cancer pain populations. Clinical practice guidelines from CPIC, NCCN, ESMO, EAPC, and ASCO were consulted for current recommendations. This narrative-critical review examines the evidence on genetic polymorphisms relevant to opioid response in cancer pain, covering three functional categories: (1) pharmacodynamic genes, including opioid receptors (OPRM1, OPRD1, OPRK1) and neuromodulatory targets (COMT, KCNJ6, MC1R); (2) pharmacokinetic enzymes (CYP2D6, CYP3A4/5, UGT2B7); and (3) membrane transporters, including ABC efflux pumps (ABCB1, ABCG2, ABCC2) and SLC uptake carriers (SLC22A1, SLCO1B1, SLC6A4, SLC6A2). A dedicated section addresses the direct and indirect interference of the tumour itself—through the tumour microenvironment, neuroimmune signalling, epigenetic reprogramming, and cancer-induced organ dysfunction—on opioid pharmacogenomics. The overall evidence is poor and fragmented, a finding intrinsic to the extraordinary diversity, large number, and context-dependent biological activity of the polymorphisms identified. The tumour itself acts as a pervasive confounder that systematically distorts genotype–phenotype relationships. The path forward requires a paradigm shift from candidate-gene association studies to system pharmacogenomics and multi-omic integration.

## 1. Introduction

Pain is among the most debilitating symptoms in cancer patients, affecting approximately 55% of those undergoing active treatment and up to 66–80% at advanced disease stages [1]. The World Health Organization (WHO) analgesic ladder, first introduced in 1986 and subsequently revised, positions opioids as the cornerstone of moderate-to-severe cancer pain management [2]. Yet clinical practice consistently demonstrates that opioid therapy is far from uniform: substantial interindividual variability exists in analgesic efficacy, opioid dose requirements, and the incidence and severity of adverse effects including nausea, constipation, sedation, cognitive impairment, and respiratory depression.

Twin studies estimate that genetic factors account for a significant proportion of the variability in opioid response, although the magnitude varies substantially depending on the phenotype measured: genetic effects explain 12–60% of the variance in pain sensitivity and analgesic response to opioid agonists, with the highest heritability observed for cold-pressor pain tolerance (49% at baseline, 60% for opioid-mediated threshold elevation) and lower estimates for heat pain responses (12%) [3,4]. This observation has driven intensive pharmacogenomic research grounded in the candidate-gene paradigm: selecting genes known or hypothesized to influence opioid pharmacodynamics or pharmacokinetics, and testing whether common polymorphisms are associated with measurable clinical outcomes. The genes investigated span the entire pharmacological pathway—from the receptor itself to the enzymes that metabolize the drug, the transporters governing bioavailability and tissue distribution, and downstream signalling molecules. Despite the scientific plausibility of this framework, accumulated evidence has been disappointing [5,6,7]. Furthermore, the tumour itself—through the microenvironment, neuroimmune signalling, metabolic reprogramming, and cancer-induced organ dysfunction—imposes a layer of biological interference that profoundly modifies both pharmacogenomic signals and opioid pharmacology in ways rarely accounted for in published studies. This review addresses both dimensions.

## 2. Opioid Biology and Pharmacogenomic Framework

### 2.1. The Endogenous Opioid System

Exogenous opioids exert their effects by binding to G protein-coupled receptors classified into three major subtypes, mu (MOR, encoded by *OPRM1*), delta (DOR, encoded by *OPRD1*), and kappa (KOR, encoded by *OPRK1*), together with the structurally related nociceptin/orphanin FQ receptor (NOP/ORL1, encoded by *OPRL1*) [8]. All clinically used analgesic opioids—morphine, oxycodone, hydromorphone, fentanyl, buprenorphine, and methadone—exert their primary analgesic action through the mu receptor. MOR activation inhibits adenylate cyclase, reduces voltage-gated calcium channel conductance, and opens inwardly rectifying potassium channels, collectively reducing neuronal excitability at supraspinal and spinal nociceptive circuits [8,9]. Agonist-induced receptor desensitization, internalization, and recycling—processes underpinning analgesic tolerance—are ligand- and context-specific. These processes are influenced by the receptor’s primary sequence and interacting proteins, providing molecular rationale for investigating receptor-coding polymorphisms.

### 2.2. Classification of Pharmacogenomically Relevant Genes

Pharmacodynamic genes encode the opioid receptors (*OPRM1*, *OPRD1*, *OPRK1*, *OPRL1*) and neuromodulatory systems gating pain perception. Variants alter drug-receptor binding affinity, receptor density, signal transduction efficiency, and the balance between analgesic and adverse effects [8,9,10].

Pharmacokinetic genes encode the cytochrome P450 enzymes (*CYP2D6*, *CYP3A4*, *CYP3A5*) and UDP-glucuronosyltransferases (*UGT2B7*) responsible for opioid biotransformation. Variants alter the rate of drug metabolism, plasma exposure to parent compounds, and the generation of pharmacologically active or toxic metabolites [5,11,12].

Membrane efflux transporter genes encode ABC-family proteins actively exporting opioids from cells and tissues, limiting CNS penetration and overall bioavailability. Key genes include *ABCB1* (P-glycoprotein), *ABCG2* (BCRP), and *ABCC2* (MRP2) [13,14].

Membrane uptake transporter genes encode solute carrier (SLC) proteins facilitating intracellular opioid entry. Key genes include *SLC22A1* (OCT1), *SLCO1B1* (OATP1B1), *SLC6A4* (SERT), and *SLC6A2* (NET), modulating hepatic uptake, neuronal distribution, and drug–drug interaction potential [15,16].

## 3. Pharmacodynamic Polymorphisms

### 3.1. OPRM1—The Mu Opioid Receptor Gene

#### 3.1.1. A118G (rs1799971): The Most-Studied Variant

The A118G SNP in exon 1 of *OPRM1* results in an asparagine-to-aspartate substitution at codon 40 (N40D), eliminating an N-glycosylation site in the extracellular N-terminal domain. The minor G allele occurs at a frequency of approximately 10–15% in European populations and up to 40–50% in East Asian populations [8,9]. In vitro studies initially reported a three-fold higher affinity of the N40D receptor for beta-endorphin, suggesting that G allele carriers might require higher doses of exogenous opioids to achieve equivalent analgesia. Clinical studies in cancer pain have yielded inconsistent results: several Scandinavian studies reported higher morphine consumption in GG homozygotes, while the large European multicentre EPOS (n = 2294) found no significant association between A118G and morphine requirements [5]. A systematic review by Klepstad et al. concluded that although A118G is biologically plausible, its clinical effect size in cancer pain is small and inconsistently demonstrated [5].

#### 3.1.2. Alternative Splicing and Isoform Diversity

OPRM1 undergoes extensive alternative splicing, generating more than 30 documented mRNA isoforms—from classical seven-transmembrane (7TM) full-length receptors to truncated six-transmembrane (6TM) variants—each with differential ligand binding, G protein coupling selectivity, and regulatory properties [9]. Intronic polymorphisms and splice-site variants influence the relative abundance of these isoforms in neuronal tissue. A patient’s net opioid response may therefore depend not only on coding SNPs but on the balance of receptor isoforms, which reflects both genetic and epigenetic regulation—a dimension largely absent from published clinical pharmacogenomic studies. Preclinical data suggest that a shift in this isoform balance toward truncated 6TM variants, which couple less efficiently to canonical G protein signalling, may attenuate morphine analgesia and has been proposed as a contributing mechanism to opioid tolerance and hyperalgesia; however, clinical confirmation that specific genetic polymorphisms drive this shift in cancer pain patients is currently lacking.

#### 3.1.3. Promoter and Regulatory Variants

Variants in the *OPRM1* promoter region, including methylation-sensitive CpG sites and transcription factor binding site polymorphisms, regulate basal gene expression and its modification by chronic pain and opioid exposure [9]. Epigenetic regulation of *OPRM1* by DNA methylation is dynamically altered by chronic opioid exposure and inflammatory states—both highly relevant to cancer pain—introducing gene–environment interactions that static genotyping cannot capture.

### 3.2. OPRD1 and OPRK1—Delta and Kappa Receptors

The delta opioid receptor (OPRD1) modulates spinal and supraspinal analgesia, particularly in neuropathic pain states. SNPs G80T (rs2234918) and T921C (rs533123) in the 3′-UTR have been associated with pain sensitivity, but studies specifically examining OPRD1 polymorphisms in cancer pain are scarce, and no variant has demonstrated clinically actionable effects [6]. The kappa receptor (OPRK1) mediates spinal analgesia, dysphoria, and sedation; the G843A promoter variant (rs6473797) and V231E coding SNP (rs1051660) have been examined in pain sensitivity models with modest associations, but clinical pharmacogenomic data in cancer pain remain preliminary [6].

### 3.3. COMT—Catechol-O-Methyltransferase

COMT encodes the enzyme degrading dopamine and norepinephrine, which modulate descending pain inhibitory pathways. The Val158Met polymorphism (rs4680) reduces enzyme thermostability and activity approximately 3–4-fold [10]. Haplotypic analysis has defined three major pain sensitivity haplotypes that predict experimental pain thresholds in healthy volunteers [10]. These haplotypes—defined by four linked SNPs (rs6269, rs4633, rs4818 and rs4680)—have been designated low pain sensitivity (LPS), average pain sensitivity (APS) and high pain sensitivity (HPS), corresponding to a gradient of COMT enzymatic activity and, consequently, of endogenous opioidergic tone [17]. In cancer pain, a landmark study by Rakvåg et al. demonstrated an association between Val158Met and morphine dose requirements in 207 patients: Met/Met homozygotes in that cohort required approximately 40% less morphine than Val/Val homozygotes (95 vs. 155 mg/24h, *p* = 0.025) [18]; a distinct COMT haplotype located in the promoter/intron 1 region—rather than the Val158Met SNP itself—has additionally been associated with a lower risk of morphine-related central side effects such as drowsiness and confusion in an independent cancer cohort [19]. Subsequent studies in larger cohorts, however, failed to replicate this finding [5,6,20]. A recent prospective study by Wong et al. (2024) in 54 advanced cancer patients found that carriers of the COMT A allele (Met) required lower opioid doses but experienced greater adverse effects, and identified novel COMT/OPRM1 genotype combinations associated with pain severity and nausea [21]. We note, however, that in the literature the reduction in morphine-related central side effects (drowsiness, confusion) has been linked to a distinct COMT haplotype located in the promoter/intron 1 region, rather than to the Val158Met (rs4680) polymorphism itself, which was not independently associated with adverse-effect risk [19]. However, the pathophysiology of cancer pain—involving peripheral sensitization, central sensitization, and tumour-mediated neuroimmune interactions—differs substantially from experimental pain models, limiting the generalizability of laboratory findings.

### 3.4. KCNJ6—Inwardly Rectifying Potassium Channel

KCNJ6 encodes the GIRK2 channel (Kir3.2), a key effector of MOR signalling. The rs2836016 variant has been associated with opioid analgesia and adverse effects in healthy volunteers and surgical patients [6]. Clinical data in cancer pain are limited to small exploratory studies requiring independent replication.

### 3.5. MC1R—Melanocortin-1 Receptor

Variants in MC1R, associated with red hair and fair skin, have been linked to altered pain sensitivity and differential responses to kappa opioid agonists in women, through the modulation of POMC-derived peptides [5]. Evidence specifically in cancer pain populations is very limited and clinical applicability remains speculative.

### 3.6. TLR4—Toll-like Receptor 4

TLR4 is an innate-immune pattern-recognition receptor expressed on spinal microglia and astrocytes, where its activation by morphine (via the MD-2 accessory protein, independently of classical mu-opioid receptor stereoselectivity) drives the glial pro-inflammatory cascade implicated in opioid-induced hyperalgesia and reduced analgesic efficacy over the course of treatment. TLR4 is also expressed on tumour cells in several malignancies, where it can modulate tumour-associated inflammation, chemoresistance and, potentially, cancer-related pain signalling, in addition to the opioid receptors already discussed. Regarding genetic polymorphism, common TLR4 SNPs (Asp299Gly, rs4986790; Thr399Ile, rs4986791) have been investigated, although rarely in the specific context of cancer pain. In the largest cancer pain cohort genotyped to date for innate-immune signalling genes (468 patients receiving transdermal fentanyl), TLR4 variants—together with TLR2 and MYD88—were not significantly associated with pain control or opioid adverse-event complaints, whereas a MYD88 variant (rs6853) was independently associated with reduced cognitive dysfunction, implicating this pathway more broadly in opioid-related morbidity even though TLR4 itself showed no clinical signal [22]. TLR4 therefore remains a biologically plausible but, on current cancer pain data, clinically unconfirmed pharmacogenomic candidate, and is included here for completeness pending further replication.

## 4. Pharmacokinetic Polymorphisms—Metabolizing Enzymes

### 4.1. CYP2D6

CYP2D6 is the primary enzyme for oxidative metabolism of codeine, tramadol, oxycodone, and hydrocodone, and contributes to oxymorphone formation. CYP2D6 is encoded by one of the most polymorphic genes in the human genome, with more than 100 functionally distinct alleles catalogued in the PharmVar database, defining four metabolizer phenotypes using the activity score (AS) system adopted by CPIC and ACMG: poor metabolizers (PM, AS = 0, ~5–10% in Europeans), intermediate metabolizers (IM, AS > 0 and ≤0.75, ~10–15%), normal metabolizers (NM, AS 1.0–2.25, ~65–80%), and ultrarapid metabolizers (UM, AS > 2.25, ~1–5% in Europeans, up to 28% in specific Ethiopian populations, attributable mainly to CYP2D6*1 × N and *2 × N gene duplications) [2,11].

The clinical implications are best established for codeine and tramadol: PMs derive no analgesic benefit from either drug, while UMs generate excess active metabolites, with documented cases of fatal respiratory depression in children and nursing infants of UM mothers—findings that have prompted regulatory label warnings from the FDA and EMA [2]. CPIC guidelines (2021) recommend avoiding both codeine and tramadol in PMs and UMs, and monitoring IMs for suboptimal response [2]. Updated CPIC recommendations issued in 2024–2025 refine this framework further, introducing more granular sub-classification of the intermediate metabolizer (IM) phenotype, revised activity score thresholds, and specific guidance for detecting copy-number variants (CNVs), which were previously prone to misclassification by standard genotyping panels and are incorporated in the present update. For hydrocodone, emerging evidence suggests clinical relevance: Reizine et al. (2021) demonstrated that oncology patients with IM/PM status treated with hydrocodone had a 5.4-fold increased risk of pain-related hospital encounters compared to NMs [23]. For morphine, fentanyl, and methadone the clinical significance of the *CYP2D6* genotype is substantially attenuated. Phenoconversion by co-medications is a critical confound addressed in Section 8.3.

### 4.2. CYP3A4 and CYP3A5

The CYP3A subfamily is responsible for the primary metabolism of fentanyl, alfentanil, sufentanil, buprenorphine, and methadone. CYP3A4 expression varies widely between individuals due to genetic, epigenetic, and environmental factors [11,12]. The CYP3A4*22 variant (rs35599367, ~5% in Europeans) is associated with reduced enzyme expression and higher oxycodone plasma concentrations in some studies. CYP3A5 is expressed significantly only in *CYP3A5*1* allele carriers; the majority of Europeans and Asians carry the loss-of-function *3 allele. Clinical evidence for CYP3A5 genotype effects on opioid outcomes in cancer pain is limited and methodologically heterogeneous [12].

### 4.3. UGT2B7—Morphine Glucuronidation

UGT2B7 generates two major morphine metabolites with opposing pharmacological profiles: morphine-6-glucuronide (M6G, potent analgesic) and morphine-3-glucuronide (M3G, pharmacologically inactive as analgesic but with neuroexcitatory properties). The M6G:M3G ratio is a key determinant of the analgesic-to-adverse-effect balance [5]. The UGT2B7 C802T polymorphism (H268Y, rs7439366, MAF ~45% in Europeans) has been associated with altered M6G:M3G ratios and more effective analgesia in some studies, but replications in cancer pain cohorts are inconsistent, in part because renal function—a major determinant of glucuronide accumulation and frequently compromised in oncology patients—is rarely controlled for adequately [5]. Because M6G is substantially more potent than morphine at the mu receptor, while M3G lacks opioid activity but retains neuroexcitatory potential, UGT2B7 genotype-driven shifts in the M6G:M3G ratio carry direct implications for both analgesic efficacy and the risk of opioid-induced neurotoxicity, particularly when renal clearance of glucuronides is impaired.

## 5. Membrane Transporter Polymorphisms

Membrane transporters govern cellular uptake and efflux of opioids at the intestinal epithelium, hepatocyte, blood–brain barrier (BBB), and neuronal membranes, profoundly influencing bioavailability, tissue distribution, and CNS penetration [13,14,15]. Two major superfamilies are pharmacogenomically relevant: the ATP-binding cassette (ABC) efflux transporters and the solute carrier (SLC) uptake transporters.

### 5.1. ABC Efflux Transporters

#### 5.1.1. ABCB1/MDR1—P-Glycoprotein

ABCB1 encodes P-glycoprotein (P-gp), an ATP-dependent efflux pump expressed at the apical surface of BBB endothelial cells, intestinal enterocytes, hepatocytes, and renal tubular cells, actively exporting morphine, loperamide, and several other opioids from the intracellular compartment [13,14]. Abcb1-knockout mouse studies unequivocally demonstrate the role of P-gp in limiting opioid brain access: knockout animals show substantially higher brain morphine concentrations and greater analgesic responses than wild-type controls [14]. Three commonly studied SNPs—C1236T (rs1128503), G2677T/A (rs2032582), and C3435T (rs1045642)—are frequently in linkage disequilibrium and analyzed as haplotypes. The synonymous C3435T variant has been proposed to affect mRNA stability and protein folding, with downstream consequences for P-gp expression, but this mechanistic link is contested: early reports assumed that this synonymous variant was itself functional, whereas subsequent work has shown that C3435T is more likely a linkage-disequilibrium proxy for the true functional variant(s) within the ABCB1 haplotype, which may explain the inconsistency of downstream clinical associations [13,14]. Clinical associations with opioid response in cancer patients have been reported for each variant, but replication is inconsistent. P-gp activity is additionally modulated by numerous chemotherapeutic agents, dexamethasone, and other oncological supportive medications, introducing drug-induced changes in P-gp function that may dwarf genotypic effects [13].

#### 5.1.2. ABCG2/BCRP—Breast Cancer Resistance Protein

ABCG2 encodes the breast cancer resistance protein (BCRP), expressed at the BBB, gastrointestinal epithelium, and placenta. The Q141K variant (rs2231142, C421A), present at a MAF of approximately 10% in Europeans and 30–35% in East Asians, is associated with reduced BCRP expression and activity [13]. Its impact on opioid disposition has been investigated primarily in in vitro and animal models; clinical data in cancer pain patients are essentially absent, although ABCG2 may become increasingly relevant as novel opioid formulations are explored.

#### 5.1.3. ABCC2/MRP2—Multidrug-Resistance-Associated Protein 2

ABCC2 encodes MRP2, expressed at the canalicular hepatocyte membrane, BBB, and intestinal enterocytes, transporting morphine glucuronides—particularly M3G—and contributing to their biliary excretion and enterohepatic recirculation. Polymorphisms −24C>T (rs717620, promoter) and 3972C>T (rs3740066) have been associated with altered MRP2 expression [18]. The −24C>T variant, associated with reduced promoter activity, could theoretically reduce M3G elimination and alter the M6G:M3G ratio—mechanistically relevant to the analgesic-to-excitatory balance of morphine. Published clinical data specifically addressing *ABCC2* polymorphisms in cancer pain are extremely sparse, representing an important unmet investigative need [18].

### 5.2. SLC Uptake Transporters

#### 5.2.1. SLC22A1/OCT1—Organic Cation Transporter 1

SLC22A1 encodes OCT1, expressed predominantly in hepatocytes where it mediates active uptake of cationic substrates—including morphine—from portal blood. Tzvetkov et al. demonstrated that OCT1 mediates hepatic morphine uptake and that OCT1-deficient subjects have reduced morphine uptake with consequentially higher plasma concentrations [20]. SLC22A1 carries several well characterized loss-of-function variants, 2 (M420del, rs35191146), 3 (R488C, rs34130495), 4 (G401S, rs34059508), and 5 (M408V, rs12208357), with a combined prevalence of ~8–10% in Europeans [20]. Subjects homozygous for loss-of-function alleles exhibit reduced hepatic morphine uptake, resulting in higher circulating morphine levels at equivalent doses. Although clinical studies in cancer pain are limited, SLC22A1 genotyping represents one of the more mechanistically coherent uptake transporter investigations to date.

#### 5.2.2. SLCO1B1/OATP1B1

SLCO1B1 encodes OATP1B1, a hepatic uptake transporter relevant to opioid pharmacogenomics through two pathways: transport of certain opioid glucuronide metabolites, and mediation of hepatic uptake of numerous co-medications in cancer patients (statins, some chemotherapeutics), potentially altering the competitive inhibition landscape for opioid transporters. The variant (c.521T>C, rs4149056) is associated with reduced transport activity. Direct effects on opioid pharmacokinetics are not well characterized, representing an area for future investigation [15].

#### 5.2.3. SLC6A4—Serotonin Transporter (SERT)

SLC6A4 encodes the serotonin reuptake transporter (SERT). The 5-HTTLPR polymorphism—a 44-base pair insertion/deletion in the promoter-linked regulatory region—generates long (L) and short (S) alleles that differ in transcriptional efficiency, with the S allele associated with reduced SERT expression [6]. Serotonergic neurotransmission is integral to descending pain inhibitory pathways from the raphe nuclei, and the serotonin system interacts with opioid circuits at multiple levels. The 5-HTTLPR variant has been associated with pain sensitivity and response to opioid analgesia, and is additionally relevant because many cancer patients receive SSRIs or SNRIs for comorbid depression or neuropathic pain, creating pharmacodynamic interactions that may modify opioid efficacy [6]. SLC6A4 is included here on this indirect pharmacodynamic rationale rather than on the strength of direct evidence linking its polymorphisms to opioid response, which to date remains weak; its discussion should therefore be regarded as exploratory.

#### 5.2.4. SLC6A2—Norepinephrine Transporter (NET)

SLC6A2 encodes the norepinephrine reuptake transporter (NET), involved in terminating noradrenergic neurotransmission. Norepinephrine is a critical mediator of descending pain inhibition through alpha-2 adrenergic receptors at the spinal level—the mechanism exploited by tramadol and tapentadol, which combine opioid receptor agonism with NET inhibition. The A457P variant (rs5569) in SLC6A2 alters NET trafficking and membrane expression [6]. Clinical studies addressing SLC6A2 polymorphisms in cancer pain are virtually absent, a significant gap given the increasing use of tramadol and tapentadol in the WHO analgesic ladder. As with SLC6A4, SLC6A2 is included on the basis of its mechanistic link to tramadol/tapentadol pharmacology rather than on direct pharmacogenomic evidence in opioid response, and should be interpreted as a hypothesis-generating inclusion pending dedicated clinical studies.

#### 5.2.5. SLCO2B1/OATP2B1 and Intestinal Absorption

SLCO2B1 encodes OATP2B1, expressed at the apical surface of intestinal enterocytes and relevant to oral drug absorption. The 935G>A variant (rs12422149) has been associated with the altered oral bioavailability of several substrates [15]. Given the substantial variability in oral morphine and oxycodone bioavailability (20–40% for morphine), intestinal transporter variants may contribute to pharmacokinetic unpredictability, but this has received minimal investigation.

## 6. Overview of Major Polymorphisms in Opioid Response

Table 1 provides a synthesized overview of the most clinically and mechanistically relevant polymorphisms, organized by functional category.

## 7. Tumour-Mediated Interference with Opioid Pharmacogenomics

A dimension systematically underappreciated in the pharmacogenomics of cancer pain is the direct and indirect interference of the tumour itself with opioid pharmacology and with the genotype–phenotype relationships that pharmacogenomic studies seek to characterize. The cancer acts simultaneously as a modifier of drug metabolism, a regulator of receptor expression, a source of neuroimmune signalling molecules, and a driver of epigenetic reprogramming—all in ways that can attenuate, amplify, or entirely negate the pharmacogenomic signal expected from a patient’s germline genotype [21,25,28]. This section addresses each of these interference mechanisms.

The mechanisms discussed below carry heterogeneous levels of evidentiary support, which we classify explicitly to avoid conflating robust findings with preliminary hypotheses. The cytokine-mediated downregulation of CYP enzymes via C/EBPβ-LIP (Section 7.2) is supported by substantial in vitro, animal, and clinical data and is consistent with the well-established phenomenon of inflammation-mediated CYP suppression; this evidence is considered relatively robust. Exosomal miRNA-mediated downregulation of OPRM1 (Section 7.3) is currently supported by a single cell/animal-based study with no independent or clinical validation and should be regarded as preliminary. Tumour-mediated modulation of membrane transporter expression (Section 7.5) rests largely on inference from chemoresistance biology rather than on direct evidence in the opioid pharmacogenomic context, and is best regarded as a plausible but largely untested hypothesis. This grading is summarized alongside the corresponding mechanistic pathway.

### 7.1. Tumour Microenvironment and Peripheral Sensitization

The tumour microenvironment (TME) generates a sustained neuroimmune milieu that profoundly alters peripheral nociceptor sensitivity and central pain processing. Tumour cells and tumour-infiltrating immune cells release a broad spectrum of algogenic and pro-nociceptive mediators—prostaglandins (particularly PGE2), bradykinin, nerve growth factor (NGF), tumour necrosis factor-alpha (TNF-α), interleukins (IL-1β, IL-6, IL-17), glutamate, ATP, endothelin-1, and hydrogen ions—that directly sensitize and activate peripheral nociceptors and contribute to cancer-induced peripheral sensitization [21,25].

The pharmacogenomic relevance is two-fold. First, the degree of peripheral sensitization is highly variable between patients and tumour types, creating a variable pharmacodynamic “set point” against which opioid effects are measured. A patient with intense TME-driven sensitization of TRPV1, TRPA1, and P2 × 3 channels in peripheral nociceptors may require substantially higher opioid doses irrespective of their OPRM1 or COMT genotype, obscuring any genotypic signal. Preclinical evidence supports this: in a murine bone cancer model, MOR expression in dorsal root ganglion neurons ipsilateral to tumour implantation was significantly reduced compared to controls (30.3% vs. 45.2% MOR-positive neuronal profiles), and intrathecal morphine was less effective than in inflammatory pain models [29]. Furthermore, cancer-derived small extracellular vesicles (sEVs) can directly sensitize nociceptors through TRPV1-dependent mechanisms and promote nascent protein translation in sensory neurons, contributing to cancer pain hypersensitivity independently of classical inflammatory mediators [1]. Specifically, HPV+ head and neck cancer-derived sEVs communicate with TRPV1+ neurons, triggering calcium influx and promoting nascent protein translation in nociceptors—a mechanism sufficient to induce pain hypersensitivity in naive mice and preventable by TRPV1 ablation [30]. In parallel, exosome-associated autotaxin-lysophosphatidic acid (ATX-LPA) signalling from fibrosarcoma cells directly sensitizes C-fibre nociceptors and dorsal root ganglion neurons, contributing to bone cancer pain through LPAR-dependent mechanisms [31]. Second, pro-inflammatory cytokines from the TME—particularly IL-6, IL-1β, and TNF-α—are potent modulators of cytochrome P450 enzyme expression, dynamically downregulating CYP3A4 and CYP2D6 activity in a manner that varies with tumour burden, disease activity, and treatment response [3,4].

### 7.2. Cancer-Induced Downregulation of Cytochrome P450 Enzymes

Systemic inflammation—a near-universal feature of advanced cancer—is associated with substantial downregulation of hepatic CYP enzyme expression. The mechanism involves cytokine-mediated suppression of the pregnane X receptor (PXR) and constitutive androstane receptor (CAR), which are the primary transcriptional activators of CYP3A4, CYP2D6, and CYP2C9 [3,5]. IL-6 in particular suppresses CYP3A4 transcription through a mechanism that, contrary to earlier assumptions, does not proceed through the JAK/STAT3 pathway. Jover et al. demonstrated that IL-6-mediated CYP3A4 downregulation requires gp130 activation but is independent of STAT3, instead proceeding through translational induction of C/EBPβ-LIP (a 20 kDa CCAAT/enhancer-binding protein β isoform lacking a transactivation domain), which competes with and antagonizes constitutive C/EBP transactivators at the CYP3A4 promoter [32]. In 3D human liver spheroid models, IL-6 and IL-1β cause near-complete (~98%) downregulation of CYP3A4, with more moderate effects on CYP2D6 [7,8]. Clinical studies in patients with advanced ovarian cancer have confirmed that CYP3A phenotypic activity is significantly repressed in the presence of tumour-associated inflammation, correlating with raised serum levels of CRP, IL-6, IL-8, and TNF-α [4].

The practical consequence for opioid pharmacogenomics is substantial: a patient who is genotypically a normal CYP3A4 metabolizer may function as a poor metabolizer with dramatically impaired fentanyl or methadone clearance when systemic inflammation is severe. Conversely, a patient with limited tumour burden may retain near-normal CYP3A4 activity. This disease-state phenoconversion—driven by the tumour itself rather than by co-medications or genetics—is dynamic, varying with disease activity, treatment response, and complications such as sepsis or acute-phase reactions [3,4]. Studies that genotype without assessing actual metabolic phenotype, or that enrol heterogeneous populations with widely varying disease burden, will exhibit profound signal dilution.

### 7.3. Tumour-Driven Epigenetic Modification of Opioid Receptor Genes

Cancer is fundamentally a disease of epigenetic dysregulation, and the epigenetic machinery reprogrammed in tumour cells has systemic ramifications that extend to non-neoplastic tissues including neurons and immune cells. Growing preclinical evidence indicates that cancer-associated epigenetic changes can alter the expression of opioid receptor genes in nociceptive neurons and in immune cells infiltrating nociceptive tissues [3,5].

*OPRM1* promoter methylation is dynamically regulated in response to chronic opioid exposure, sustained inflammation, and neuroimmune interactions generated by the TME. Hypermethylation of the *OPRM1* CpG island—observed in some tumour-bearing animal models and in peripheral blood mononuclear cells of cancer patients—reduces receptor expression and could contribute to apparent opioid tolerance or resistance that is epigenetic in origin rather than pharmacokinetic. Histone deacetylases (HDACs) and DNA methyltransferases (DNMTs) with altered activity in the cancer setting can modify chromatin accessibility at *OPRM1*, *COMT*, and transporter gene promoters, generating discordance between germline genotype and functional gene expression that static genotyping is unable to detect [5].

Furthermore, tumour-derived exosomes carry microRNAs (miRNAs) that can regulate gene expression in distant tissues, including neurons. Li et al. demonstrated that lung-cancer-cell-derived exosomal let-7d-5p is taken up by dorsal root ganglion (DRG) neurons and directly inhibits the protein level of OPRM1, contributing to the generation and maintenance of cancer-induced bone pain in a murine model [33]. This finding provides direct experimental evidence that tumour-secreted exosomal miRNAs can downregulate opioid receptor expression in nociceptive neurons, introducing a novel layer of tumour-driven pharmacological interference that operates post-transcriptionally and independently of germline genotype [3,9].

Beyond exosomal miRNA, a broader landscape of non-coding RNA regulation is increasingly implicated in opioid signalling and nociceptive sensitization. Long non-coding RNAs (lncRNAs) and circular RNAs (circRNAs) can act as molecular “sponges” within competing endogenous RNA (ceRNA) networks, sequestering microRNAs that would otherwise regulate OPRM1 and other nociceptive-pathway genes, thereby modulating receptor expression indirectly. These mechanisms have been characterized predominantly in preclinical models of neuropathic and inflammatory pain; their relevance to cancer pain and opioid pharmacogenomics specifically remains an emerging area, and their potential modulation by the tumour microenvironment itself warrants dedicated investigation.

### 7.4. Cancer-Induced Organ Dysfunction and Pharmacokinetic Distortion

Hepatic and renal dysfunction, common in patients with advanced or metastatic cancer, fundamentally alter opioid pharmacokinetics irrespective of genotype. Hepatic metastases—particularly in colorectal, breast, and gastric cancers—and cancer-related biliary obstruction impair first-pass morphine metabolism and reduce UGT2B7 and CYP3A4 functional capacity. The result is elevated plasma morphine concentrations, disproportionate M6G accumulation, and unpredictably prolonged drug half-lives.

Cancer-related renal dysfunction—from direct tumour invasion, ureteric obstruction, dehydration, nephrotoxic chemotherapy, or paraneoplastic glomerulonephritis—impairs the renal elimination of morphine glucuronides. Accumulation of M6G in renal failure has well-documented clinical consequences, including prolonged and profound opioid effect, opioid-induced hyperalgesia from M3G accumulation, and respiratory depression. These organ-dysfunction-driven effects on opioid pharmacokinetics greatly exceed the effect sizes of most pharmacogenomic variants, yet they are rarely incorporated as covariates in pharmacogenomic analyses.

Hypoalbuminemia—prevalent in cancer cachexia and malnutrition—reduces plasma protein binding of opioids such as fentanyl and buprenorphine (which are highly protein-bound), increasing the free fraction of drug available for CNS penetration. This protein-binding effect is a pharmacokinetic variable of clinical significance that interacts with transporter genotype: a patient with *ABCB1* variants conferring reduced P-gp activity and concurrent hypoalbuminemia may have substantially higher effective CNS opioid exposure than either factor alone would predict.

### 7.5. Tumour-Related Modulation of Transporter Expression

Beyond systemic inflammation-mediated CYP downregulation, tumours and their microenvironment can directly alter the expression of opioid-relevant membrane transporters. Hepatic metastases can disrupt the zonated expression of OAT/OCT transporters in hepatocyte populations, compromising hepatic opioid uptake in ways that are not captured by germline *SLC22A1* genotyping [10,11]. Tumour-associated macrophages and myeloid-derived suppressor cells (MDSCs) express altered profiles of ABC transporters including ABCB1, which regulates their cytokine secretion and drug efflux capacity, potentially influencing local opioid concentrations in inflamed tissues [12,13].

Chemotherapy agents commonly used in oncology exert collateral effects on transporter expression. Taxanes (paclitaxel, docetaxel) induce ABCB1 expression through PXR activation, potentially reducing opioid CNS penetration during chemotherapy cycles [11,14]. Oxycodone itself has been shown to induce P-gp overexpression (1.3–4.0-fold) in a rat model, with consequent reduction in paclitaxel tissue distribution by 38–90%, demonstrating bidirectional opioid–chemotherapy transporter interactions [34]. Platinum compounds (cisplatin, oxaliplatin) cause renal tubular injury that impairs organic cation transporter function, altering the elimination of OCT substrates including morphine. Vinca alkaloids and anthracyclines are substrates and inhibitors of P-gp, creating dynamic drug–drug–gene interactions that are virtually impossible to predict without real-time pharmacokinetic monitoring [10,14].

### 7.6. Cancer-Induced Pain Mechanism Shift and Pharmacodynamic Interference

The mechanism of pain in cancer patients is not static: it evolves with disease progression, treatment, and the development of complications. A patient may transition from predominantly nociceptive bone pain (opioid-responsive) to a mixed nociceptive–neuropathic syndrome from perineural invasion (partially opioid-resistant) to predominant neuropathic pain from chemotherapy-induced peripheral neuropathy (opioid-poorly responsive) over the course of their illness [2,9].

Perineural invasion by tumour—a common finding in pancreatic, colorectal, and head-and-neck cancers—induces ectopic discharge and sensitization of primary afferent neurons through direct nerve injury and the release of NGF, Sonic Hedgehog, and other neurotrophic factors. The resulting altered nociceptor phenotype, including upregulation of Nav1.7, Nav1.8, and TRPV1, fundamentally changes the receptor context in which opioids exert their effect [9]. Recent evidence further demonstrates that tumour-infiltrating nociceptor neurons actively promote immunosuppression through recruitment of MDSCs and induction of CD8+ T cell exhaustion, establishing a bidirectional neuroimmune axis within the TME that extends beyond pain modulation to influence tumour biology itself [35]. Central sensitization driven by sustained nociceptive input from the tumour—manifested clinically as allodynia, hyperalgesia, and opioid-refractory pain—creates a pharmacodynamic environment where the expected genotype-to-analgesic-response relationship predicted from preclinical models or non-cancer pain studies is systematically distorted [2,9].

### 7.7. Implications for Pharmacogenomic Research Design

The tumour interference mechanisms described above have direct and profound implications for the design of pharmacogenomic studies in cancer pain. Failure to control for systemic inflammation (measured by CRP, IL-6, or acute-phase protein profiles), renal and hepatic function, current chemotherapy and its transporter-modulating effects, disease stage and tumour burden, and pain mechanism will result in studies with grossly inflated residual variance—reducing statistical power to detect genuine pharmacogenomic associations and generating non-replicable findings [3,18,20].

Future studies should incorporate biomarkers of TME activity, epigenetic profiling of key opioid system genes in accessible tissues (whole blood, buccal cells), and phenotypic metabolic assessments using validated probe substrates alongside genotyping. Statistical models must include tumour-related covariates and should apply mixed-effects frameworks capable of accommodating the time-varying nature of disease-related interference. Only through such rigorous designs can genuine pharmacogenomic signals be disentangled from the dominant tumour-driven background noise that has obscured them in the majority of published studies [18,20,21].

### 7.8. Gut Microbiome-Mediated Interference

A further, still under-explored layer of tumour-associated interference operates through the gut microbiome. Bacterial β-glucuronidase activity can deconjugate morphine glucuronides (including M3G and M6G) within the intestinal lumen, altering enterohepatic recirculation and effective systemic exposure, while microbial modulation of bile acid metabolism affects opioid pharmacokinetics more broadly. On the pharmacodynamic side, the gut–brain axis and microbiota-dependent immune modulation may influence neuroinflammation and nociceptive sensitivity. In oncology patients, chemotherapy, radiotherapy, and antibiotic exposure profoundly and repeatedly perturb the microbiome, introducing an additional, dynamic source of interindividual variability in opioid response that compounds, and may partly overlap with, the tumour microenvironment-driven mechanisms described above.

## 8. Critical Analysis of the Evidence

### 8.1. Methodological Limitations

The literature on opioid pharmacogenomics in cancer pain is characterized by pervasive methodological weaknesses. The most critical is inadequate sample size: the majority of published studies recruited 50–300 patients, providing insufficient statistical power to detect small-to-moderate genetic effects, particularly for variants with low minor allele frequencies [18,20]. Outcome heterogeneity constitutes a second major challenge: studies variously use total opioid dose, dose change over fixed periods, pain intensity scores (NRS, VAS, BPI), number of breakthrough doses, or adverse-effect frequency as primary endpoints—outcomes that are not interchangeable and may be under distinct genetic regulation [21]. The lack of standardization in calculating morphine equivalent daily dose (MEDD), with variable conversion factors between studies, introduces an additional source of heterogeneity. Confounding variables—renal and hepatic function, concurrent medications, cancer type and stage, pain mechanism, psychological comorbidities, and prior opioid exposure—are inconsistently controlled. In cancer patients, these confounders often carry greater effect sizes than the genetic variants under investigation.

### 8.2. Population Stratification and Ethnic Heterogeneity

Genetic polymorphism frequencies vary substantially across populations of different ancestral origins. For *OPRM1* A118G, the G allele frequency is 10–15% in Europeans but 38–48% in East Asians. CYP2D6 poor metabolizer prevalence ranges from ~5–10% in Europeans to <1% in Asian populations but exceeds 20% in some sub-Saharan African populations [25]. *ABCG2* Q141K is rare (<5%) in Africans but common in Asians (~30%). Studies conducted in ethnically homogeneous populations may not be generalizable, and meta-analyses pooling studies from diverse ethnic backgrounds without stratification conflate biological differences with statistical noise.

A systematic, population-stratified view (gnomAD/1000 Genomes Project) further clarifies where testing is likely to be cost-effective. OPRM1 A118G ranges from roughly 10–15% in Europeans to 40–50% in East Asians, with intermediate frequencies reported in South Asian and Latin American populations and generally lower frequencies in sub-Saharan African populations. CYP2D6 poor metabolizer frequency is highest in Europeans (5–10%) and essentially absent (<1%) in East Asians, whereas ultrarapid-metabolizer alleles driven by CYP2D6 gene duplications are most prevalent in East African and parts of Mediterranean populations. UGT2B7 C802T and ABCB1 C3435T also show substantial inter-ancestry variation. These differences carry direct implications for implementation: CYP2D6-guided avoidance of codeine/tramadol is more likely to be cost-effective where PM/UM frequencies are clinically meaningful (e.g., European and East African populations) but offers limited yield in East Asian populations where PM is rare, whereas OPRM1 A118G-based strategies may be comparatively more informative in East Asian cohorts. It must also be acknowledged as a major limitation of the field that the large majority of opioid pharmacogenomic studies, including most of those reviewed here, have been conducted in populations of European ancestry; data from African, South Asian, and Latin American populations remain critically scarce, constraining the global generalizability of the associations summarized in this review.

### 8.3. Phenoconversion

Phenoconversion—discordance between genotypic and phenotypic metabolizer status caused by drug–drug interactions, disease states, or nutritional factors—is among the most clinically important but least-addressed confounds in cancer pain pharmacogenomics. Cancer patients are among the most polypharmacy-burdened patient populations. CYP2D6 can be effectively inhibited to PM levels by antidepressants (paroxetine, fluoxetine), antipsychotics, and some targeted therapies. CYP3A4 is strongly induced by dexamethasone and some anticonvulsants, and inhibited by azole antifungals [3,6,25]. Additionally, as discussed in Section 7.2, the tumour-driven inflammatory downregulation of CYP enzymes constitutes a further mechanism of phenoconversion that is pathophysiological rather than drug-induced. Studies relying on germline genotyping alone will systematically misclassify a substantial proportion of patients.

The clinical significance of phenoconversion is underscored by the ADOPT PGx trial, a multicentre randomized controlled trial of 1602 participants undergoing surgery. CYP2D6-guided prescribing recommendations—including avoidance of hydrocodone, tramadol, and codeine in PMs, IMs, and UMs—significantly altered prescribing patterns (concordance 64% vs. 27% in usual care), yet did not improve postoperative pain control compared to standard care [36]. This negative result in a controlled surgical setting, where confounders are fewer than in cancer pain, highlights the challenge of translating genotype information into measurable clinical benefit and supports the argument that opioid response is determined by a complex interplay of factors that extends well beyond the CYP2D6 genotype.

### 8.4. Epistasis and Gene–Environment Interactions

The analgesic response to opioids emerges from a complex network of gene–gene interactions (epistasis) and gene–environment interactions that cannot be captured by analyzing individual SNPs in isolation [21]. A patient who is an *ABCB1* C3435T minor allele carrier, an OCT1 LOF homozygote, and a CYP2D6 poor metabolizer will have a phenotype non-linearly determined by this combination—not predictable from knowledge of any single variant. Gene–environment interactions are particularly pronounced in oncology: the TME, systemic inflammation, cachexia, and chemotherapy neurotoxicity dynamically alter the pharmacodynamic landscape in ways that evolve with disease progression and treatment response. In a two-stage cross-sectional study of 1027 Chinese cancer patients genotyped for 110 SNPs, no significant single-SNP or haplotype associations with opioid response after multiple-testing correction were found, but demonstrated that multidimensional gene–environment interaction models could explain opioid response variability—a finding that underscores the inadequacy of single-variant approaches [37].

### 8.5. GWAS Evidence

The largest candidate-gene association study in cancer pain—the European Pharmacogenomics of Opioids Study (EPOS), comprising 2294 patients from multiple European centres—tested 112 SNPs in 25 candidate genes and found no significant associations with opioid dose in both development and validation samples after appropriate correction [29]. This negative result is profoundly informative: it suggests that individual common variants in established candidate genes explain at most a small fraction of opioid response variability in cancer patients, and that a purely SNP-centric candidate-gene approach is insufficient.

This negative result also warrants interpretation in light of broader statistical and methodological phenomena beyond sample size and outcome heterogeneity alone. First, publication bias and the “winner’s curse” mean that positive associations from small, underpowered candidate-gene studies are disproportionately likely to be published, systematically inflating apparent effect sizes in the early literature; the failure of larger, well-powered cohorts such as EPOS to replicate these findings is, at least in part, the expected statistical consequence of this bias rather than definitive proof of no genetic contribution. Second, standard SNP arrays and candidate-gene panels capture predominantly common variants (minor allele frequency >1%); rare variants and structural variants (copy-number variants, indels) may carry larger individual effect sizes but are missed entirely by these platforms, and whole-genome sequencing with burden-testing approaches may be required to uncover this hidden component of the genetic architecture. Third, a variant may show no direct association with a clinical phenotype while still influencing gene expression through regulatory mechanisms (expression quantitative trait loci, eQTLs); integrating GWAS findings with transcriptomic resources such as GTEx is increasingly recognized as necessary for correctly interpreting pharmacogenomic associations and represents an important direction for future research in this field.

However, the EPOS was not a genome-wide association study (GWAS) in the strict sense. True GWAS approaches in cancer pain have begun to yield results, albeit with modest sample sizes. Beyond studies conducted specifically in cancer pain cohorts, larger general-population biobank analyses provide complementary context. Ferreira et al. (2020) analyzed chronic pain across more than 380,000 UK Biobank participants and identified numerous risk loci, with pathway enrichment implicating oligodendrocyte differentiation, neuronal guidance, and endolysosomal function; a subsequent 2024 meta-analysis extended this locus discovery further [38] Although these studies did not use opioid response as the outcome, they delineate the polygenic architecture of pain susceptibility that plausibly interacts with the opioid-specific pharmacogenomic variants discussed in this review, and support polygenic rather than single-gene models of individual variability. Nishizawa et al. (2022) conducted a GWAS in 428 cancer pain patients and identified two intronic SNPs (rs1283671 and rs1283720) in the *ANGPT1* gene (encoding angiopoietin-1) that reached genome-wide significance (*p* < 5.0 × 10^−8^) for association with average daily opioid requirements [39]. More recently, Minnai et al. (2025) performed a GWAS in 2057 European advanced cancer patients treated with opioids and identified five non-coding variants on chromosome 20, intronic to *PCMTD2* and downstream of *OPRL1* (the nociceptin receptor gene), that reached genome-wide significance for association with pain intensity; these variants act as expression quantitative trait loci modulating *PCMTD2* and *OPRL1* expression [40]. These emerging GWAS findings suggest that novel, previously unsuspected loci may contribute to opioid response in cancer pain, but require replication in independent cohorts. The overall GWAS landscape is consistent with a highly polygenic architecture—hundreds to thousands of variants each contributing small effects—and also raises the possibility of significant contributions from rare variants, structural variants, and copy-number variants not captured by standard SNP arrays.

## 9. Diversity of Polymorphisms as Root Cause of Poor Evidence

### 9.1. The Number of Variants

The catalogue of pharmacogenomically relevant polymorphisms in the opioid system is vast. *OPRM1* alone harbours more than 150 catalogued variants. *CYP2D6* has over 100 functionally distinct star alleles [25]. Adding *ABCB1*, *SLC22A1*, *SLCO1B1*, *UGT2B7*, *COMT*, and novel GWAS loci, the space of potentially relevant variants numbers in the thousands. Testing hundreds of variants in underpowered samples generates thousands of association tests; early positive findings in small samples are regressed to the mean in larger replication cohorts. The replication failure characterizing the opioid pharmacogenomics literature is at least partly a consequence of this multiple-testing burden [18,20,21].

### 9.2. Functional Heterogeneity

Variants span at least five mechanistic classes within any single gene: missense variants altering protein structure; synonymous variants affecting co-translational folding or mRNA stability; UTR variants modulating translational efficiency; promoter variants affecting transcription factor binding; and intronic variants regulating splice-site selection. Each class has distinct functional implications and different effect sizes, yet all are routinely categorized as “polymorphisms” in the literature without functional stratification [8,26]. Without experimental characterization of individual variants in relevant cell systems, their inclusion in clinical association studies rests on positional candidate assumptions rather than mechanistic evidence.

### 9.3. Context-Dependent Biological Activity

The biological activity of any given polymorphism is not intrinsic to that variant in isolation—it depends critically on the cellular and systemic context. *OPRM1* promoter methylation is dynamically regulated by opioid exposure and inflammation; a variant augmenting receptor expression under baseline conditions may have no net effect if regulatory elements are epigenetically silenced by cancer-associated reprogramming [5,9]. In cancer patients, the context is systematically distorted relative to the healthy-volunteer or stable chronic pain populations informing prior assumptions: systemic inflammation, cachexia, hypoalbuminemia, tumour-induced sensitization, and chemotherapy neurotoxicity create an environment where expected genotype–phenotype relationships may be attenuated, amplified, or reversed. This context-dependence renders direct translation of pharmacogenomic findings between populations epistemically hazardous.

### 9.4. Combinatorial Complexity

If we conservatively assume twenty pharmacogenomically relevant genes with an average of five common variants each, the number of possible multi-locus genotype combinations is ∏i = 12,035 = 3100 ≈ 5.15 × 10^47^—a number that vastly exceeds any conceivable clinical cohort. Even restricting attention to the ten most-studied genes and their major functional alleles, the number of distinct genetic profiles in a population of 2000 patients is so large that most profiles will be represented by a handful of individuals. Traditional statistical approaches require sufficient numbers of individuals with a specific genotype to power an association test—a criterion unachievable for most multi-locus combinations in any existing cohort [21]. Machine learning approaches offer potential solutions, but require training datasets far larger than any existing cancer pain pharmacogenomics cohort, and their predictions require prospective validation before clinical implementation.

## 10. Clinical Implications and Future Perspectives

### 10.1. Current Recommendations

Major clinical guidelines for cancer pain management do not recommend routine pharmacogenomic testing prior to opioid prescribing for most opioids and polymorphisms. However, the positions of individual societies differ in nuance. NCCN guidelines (v1.2026) state that pharmacogenomic testing “may be considered” prior to initiation or during analgesic pharmacologic treatment when concerns of toxicity or lack of analgesic response are demonstrated or suspected, and provide specific recommendations for CYP2D6 testing in the context of codeine, tramadol, and hydrocodone prescribing, as well as CYP2C19/CYP2D6 testing for adjuvant analgesics (amitriptyline, doxepin) and CYP2C9 testing for NSAIDs (celecoxib, meloxicam, ibuprofen) [27]. ASCO guidelines [41] state that “there is insufficient evidence to recommend for or against” CYP2D6 testing to guide opioid selection—a position of neutrality rather than recommendation against testing. EAPC recommendations [42] do not include pharmacogenomic testing in their evidence-based framework.

The single exception with sufficient evidence for regulatory action is CYP2D6 testing in specific high-risk scenarios before codeine and tramadol prescription, particularly in pediatric populations and lactating mothers, as codified in CPIC guidelines [26]. For all other opioids and polymorphisms, clinical practice remains empirical, guided by careful dose titration, monitoring, and rotation when response is inadequate or toxicity occurs.

### 10.2. The Pharmacome and Multi-Omic Integration

The most promising conceptual framework may be the “pharmacome”: integrated, multi-omic characterization of a patient’s pharmacological phenotype incorporating genomics, transcriptomics, epigenomics, proteomics, and metabolomics [21]. Pilot studies integrating urinary metabolomic profiles with genomic data have identified composite patterns predictive of opioid response that neither modality captures alone. Critically, the pharmacome concept explicitly accommodates tumour-driven interference by measuring functional molecular states rather than relying solely on germline sequence. Yennurajalingam et al. (2021), in a prospective study of 174 advanced cancer patients, demonstrated that gene-block (SKATO) analysis incorporating multiple variants within neuroimmune pathway genes (*CXCL8*, *IL-6*, *STAT6*) was significantly associated with pain severity and opioid response, suggesting that pathway-level rather than single-variant analysis may better capture pharmacogenomic signals in cancer pain [43].

### 10.3. AI and Network-Based Approaches

Network pharmacology and artificial intelligence offer tools for modelling multi-gene, multi-variant complexity without requiring explicit specification of all pairwise interactions [21]. Federated learning approaches, which allow models to be trained across multiple institutions without centralizing sensitive patient data—in practice, multiple hospitals jointly train a shared predictive model by exchanging only statistical model updates rather than individual patient records, preserving data privacy while still combining the statistical power of multicentre cohorts—may overcome sample size barriers that have limited pharmacogenomic discovery. Deep phenotyping of opioid response leveraging digital health tools, electronic prescribing data, patient-reported outcomes, and wearable biosensors will be essential for generating high-quality, large-scale datasets. A recent large-scale GWAS meta-analysis of prescription analgesic use across UK Biobank and FinnGen identified 140 genetic associations with chronic pain phenotypes, including 78 novel loci, and implicated oligodendrocyte differentiation, neuronal guidance, and endolysosomal function—demonstrating the power of large biobank-based approaches to uncover pain-relevant biology [44].

### 10.4. Incorporating Tumour Interference in Clinical Decision Tools

Future clinical pharmacogenomic decision support tools for opioid prescribing in cancer pain must explicitly integrate disease-related variables alongside genotype information: inflammatory biomarkers (CRP, IL-6, albumin), organ function indices (GFR, Child–Pugh score), current chemotherapy regimen and its known transporter and enzyme interactions, and pain mechanism assessment. A genotype report interpreted without these covariates is of limited clinical utility and may be misleading in a proportion of patients. The development and prospective validation of such integrated decision tools should be a priority for future research [3,4].

## 11. Conclusions

Opioid pharmacogenomics in cancer pain is a scientifically compelling field that has not yet delivered on its clinical promise. Despite substantial research effort spanning pharmacodynamic genes (*OPRM1*, *COMT*, *KCNJ6*), pharmacokinetic enzymes (*CYP2D6*, *CYP3A4/5*, *UGT2B7*), ABC efflux transporters (*ABCB1*, *ABCG2*, *ABCC2*), and SLC uptake transporters (*SLC22A1*, *SLCO1B1*, *SLC6A4*, *SLC6A2*), the aggregate evidence base remains weak, contradictory, and poorly replicable [18,20,21].

A critical and novel dimension addressed in this review is the direct interference of the tumour with opioid pharmacogenomics. Through TME-driven peripheral sensitization, cytokine-mediated CYP enzyme downregulation via C/EBPβ-LIP and related transcriptional mechanisms, epigenetic reprogramming of opioid receptor genes, exosomal miRNA-mediated downregulation of OPRM1 in nociceptive neurons, cancer-induced organ dysfunction, tumour-modulated transporter expression, and dynamic shifts in pain mechanism, the tumour acts as a pervasive confounder that systematically distorts the genotype–phenotype relationships that pharmacogenomic studies seek to characterize [1,2,3,4,5,6,9]. This interference is not a marginal phenomenon but a central feature of the biology, rendering the oncology setting fundamentally more complex than the stable chronic pain or healthy-volunteer populations from which much of our prior pharmacogenomic understanding is derived.

The weakness of the evidence reflects both methodological inadequacies and the intrinsic complexity of the system: the analgesic response to opioids is a highly polygenic, multifactorial trait shaped by hundreds of variants across dozens of genes, interacting within networks of formidable epistatic and environmental complexity. The diversity of variants in number, molecular mechanism, functional penetrance, and context-dependency generates a combinatorial landscape that the candidate-gene single-SNP paradigm is fundamentally unable to resolve [21].

The path forward requires a paradigm shift: from candidate-gene association studies to system pharmacogenomics; from genotyping to multi-omic pharmacome characterization; from underpowered single-centre studies to federated international consortia; and from static genotype reports to dynamic, AI-assisted clinical decision support tools integrating genetic, tumour-biological, pharmacokinetic, and patient-reported data. Until this transition is achieved, cancer pain management will remain a predominantly empirical discipline—guided by clinical judgement, careful monitoring, and therapeutic flexibility—with pharmacogenomics contributing at the margins rather than transforming the therapeutic paradigm [18,20,21].

## Figures and Tables

**Table 1:** Key polymorphisms in opioid pharmacogenomics. MAF = minor allele frequency, approximate values for European populations unless otherwise stated. LOF = loss-of-function; M6G = morphine-6-glucuronide; M3G = morphine-3-glucuronide; PM = poor metabolizer; UM = ultrarapid metabolizer.

Gene/Protein	Variant (rsID)	MAF	Functional Mechanism	Evidence in Cancer Pain	References
OPRM1 (MOR)	A118G/N40D (rs1799971)	10–50% G	Alters glycosylation; receptor kinetics	Weak; inconsistent	[5,8,9]
OPRM1 (MOR)	Splice variants (multiple)	Variable	Isoform-dependent receptor pharmacology	Preclinical only	[9]
OPRM1 (MOR)	Promoter CpG methylation	Epigenetic	Regulates basal receptor expression	Preclinical	[9,24]
OPRD1 (DOR)	G80T, T921C	5–15%	Receptor expression; splicing effects	Preliminary	[6]
OPRK1 (KOR)	G843A promoter, V231E	10–20%	Promoter activity; receptor structure	Preliminary	[6]
COMT	Val158Met (rs4680)	~50% Met	3–4× reduced enzyme thermostability	Weak; confirmed in one major study, not replicated in larger cohorts	[5,6,10,18,21]
KCNJ6 (GIRK2)	rs2836016	~40%	K+ channel effector of MOR signalling	Preliminary	[6]
MC1R	Multiple (R151C, etc.)	15–40%	POMC peptide modulation; KOR sensitivity	Preclinical	[25]
CYP2D6	PM alleles (4,5,6,41)	5–10% PM	Absent/reduced codeine/tramadol → active metabolite conversion	Strong for codeine and tramadol; Moderate for hydrocodone	[11,23,26]
CYP2D6	UM alleles (2xN, 35)	1–5% UM	Ultrarapid metabolism; toxicity risk	Strong for codeine/tramadol fatalities	[26]
CYP3A4	22 (rs35599367)	~5%	Reduced enzyme expression	Preliminary; oxycodone AUC data	[11,12]
CYP3A5	3 (rs776746)	~50–90%	Loss of CYP3A5 expression	Weak; masked by CYP3A4	[12]
UGT2B7	C802T/H268Y (rs7439366)	~45% T	Altered M6G:M3G ratio	Weak; renal function confounds	[13,27]
ABCB1 (P-gp)	C3435T (rs1045642)	~55% T	Reduced P-gp; possible increased CNS entry	Weak; mechanism disputed	[14,15]
ABCB1 (P-gp)	G2677T/A (rs2032582)	~50% T/A	Haplotype effects on P-gp expression	Weak; inconsistent	[14]
ABCG2 (BCRP)	Q141K/C421A (rs2231142)	10–35%	Reduced BCRP expression/activity	Preclinical only for opioids	[14]
ABCC2 (MRP2)	−24C>T (rs717620)	~20%	Reduced promoter activity; reduced M3G export	Preliminary; mechanistically relevant	[27]
SLC22A1 (OCT1)	2,3,4,5 (multiple rs)	~8–10% LOF	Reduced hepatic morphine uptake; higher plasma levels	Moderate	[20]
SLCO1B1 (OATP1B1)	5/c.521T>C (rs4149056)	~15%	Reduced hepatic organic anion uptake	Preliminary; indirect relevance	[16]
SLC6A4 (SERT)	5-HTTLPR ins/del	~40% S	Reduced SERT expression	Preliminary	[6]
SLC6A2 (NET)	A457P (rs5569)	~15%	Altered NET trafficking/expression	Absent	[6]
SLCO2B1 (OATP2B1)	935G>A (rs12422149)	~10%	Reduced intestinal oral drug absorption	Absent	[16]

Note: the letter(s) following the percentage in the MAF column (e.g., G, T, T/A, PM, UM, Met, S, LOF) denote the specific allele, genotype, or metabolizer status to which that frequency refers, and are not citations from the literature. Evidence levels: Strong = replicated clinical data with guideline-level recommendations; Moderate = mechanistic support with limited clinical replication; Weak = inconsistent or contradictory clinical data; Preliminary = small studies requiring replication; Preclinical = in vitro or animal data only; Absent = no published data in cancer pain populations.

## Data Availability

No new data were created or analyzed in this study. Data sharing is not applicable to this article.
